# Clinical characteristics and long-term management for patients with vitamin D-dependent rickets type II: a retrospective study at a single center in Saudi Arabia

**DOI:** 10.3389/fendo.2024.1365714

**Published:** 2024-05-30

**Authors:** Afaf Alsagheir, Abdullah Al-Ashwal, Amal Binladen, Raghad Alhuthil, Faisal Joueidi, Khushnooda Ramzan, Faiqa Imtiaz

**Affiliations:** ^1^ Section of Pediatric Endocrinology, Department of Pediatrics, King Faisal Specialist Hospital and Research Centre, Riyadh, Saudi Arabia; ^2^ College of Medicine, Alfaisal University, Riyadh, Saudi Arabia; ^3^ Department of Clinical Genomics, Centre of Genomic Medicine, King Faisal Specialist Hospital and Research Centre, Riyadh, Saudi Arabia

**Keywords:** HVDD type II, hereditary vitamin D rickets type II, growth retardation, growth velocity, vitamin D, alopecia

## Abstract

**Introduction:**

Hereditary Vitamin D-dependent rickets type II (HVDDR-type II) is a rare autosomal recessive disorder caused by molecular variation in the gene encoding the vitamin D receptor (VDR). This study aims to evaluate phenotype and genotype characteristics and long-term follow-up of the largest group of patients with (HVDDR-type II) in Saudi Arabia.

**Methodology:**

We conducted a retrospective chart review to collect the clinical, biochemical, and genetic data for all HVDDR-type II patients currently receiving treatment at King Faisal Specialist Hospital & Research Centre, Riyadh, Saudi Arabia.

**Results:**

A total of 42 patients, 57.1% female, and 42.9% male were included in the study. Seven patients were treated with high doses of oral calcium, while 35 patients were treated with IV calcium infusion. The median age at presentation was 15.5 months. Alopecia was found in 97.6%, 21.4% presented with bowing legs, 14.3% with delayed walking, 9.5% with seizure, and 2.4% presented with respiratory failure, while a family history of the disease was positive in 71.4% of total patients. Molecular genetic testing of the VDR gene in our cohort identified six different gene variants c.885 C>A (p.Tyr295Ter), c.88 C>T (p.Arg30Ter), c.1036G>A (p.Val346Met), c.820C>T (p.Arg274Cys), c.803 T>C (p.Ile268Thr), and c.2T>G (p.Met1?).

**Conclusion:**

We are describing the largest cohort of patients with HVDDR-type II, their clinical biochemical findings, and the most prevalent genetic variants in our population.

## 1 Introduction

Hereditary vitamin D-resistant rickets (HVDRR), also referred to as Hereditary vitamin D-dependent type II rickets (HVDDR-II), is a rare autosomal recessive disorder caused by molecular variation in the gene encoding the vitamin D receptor (*VDR*). HVDDR-II is characterized by early onset of rickets presentation and biochemical parameters such as low serum calcium levels, high parathyroid hormone, high alkaline phosphatase, and high 1,25(OH)_2_D levels (1).

Several types of variants have been identified in the *VDR* gene as the leading cause of HVDDR-II, such as non-sense, substitutions, insertions, duplications, deletions, and splice-site (2). The primary defect caused by the mutant *VDR* gene is a decrease in intestinal calcium and phosphate absorption, which leads to decreased bone mineralization and rickets (3), despite high levels of 1,25(OH)_2_D (4). Patients with HVDDR-II have rickets along with hypocalcemia, hypophosphatemia, secondary hyperparathyroidism, and elevated serum 1,25(OH)_2_D levels, which is a hallmark for diagnosing this disease. The patient will present with the same characteristic features of nutritional rickets in the form of frontal bossing, bowing of the leg, growth failure, dental caries, and enamel hypoplasia (5).

The treatment of HVDDR-II is not standardized in most cases. Studies have shown that oral calcium supplements can improve patients’ clinical features. However, high doses are required to achieve therapeutic results. IV calcium is proven more potent and effective in improving clinical, radiological, and biochemical outcomes in a short period (2, 6–8). This can significantly improve the levels of calcium and phosphate in the first period of infusion, followed by improvement of alkaline phosphatase and parathyroid hormones.

This study presents the clinical, biochemical and genotype characteristics, long-term outcomes, and response to treatment in one of the most extensive patient series with HVDDR-II with variation in the *VDR* gene.

## 2 Materials and methods

### 2.1 Patient cohort

Forty-two patients diagnosed with HVDDR-type II, aged 18 years and less, with Homozygous *VDR* gene variants, who attended the pediatric endocrine clinic at King Faisal Specialist Hospital and Research Center (KFSH&RC), Riyadh.

Information regarding demographics, initial clinical presentation, family history, phenotype, growth parameters, biochemical data at presentation, and genetic variation were retrospectively extracted and analyzed. Follow-up data reporting the most recent clinical data, growth status, treatment, and outcome were also collected.

### 2.2 Clinical evaluation

The patients were interviewed on each visit for their symptoms related to hypocalcemia (e.g., carpopedal spasms, seizures, and fractures). They were examined for signs of rickets (e.g., bowing, wide wrist, rachitic rosary). Compliance with the treatment was measured and assessed by interviewing the family on every visit. Patients were considered/declared non-compliant in case of a missed dose more than 2 times weekly in 3 months or more. Growth status during follow-up was assessed by measurement of the height/length age–gender standard deviation score (SDS) in the Saudi population (9). A height SDS below –2 was considered short stature, and below -3 SDS was severely short.

Every month, serum calcium (Ca), phosphorus (PO_4_), Intact parathyroid hormone (iPTH) (measured by electrochemiluminescence immunoassay “ECLIA”), and alkaline phosphatase (ALP), spot urine calcium/creatinine ratio are measured. Every 3 months, radiological studies (X-rays of the left hand and knee for signs of rickets) were done. Additionally, a renal ultrasound is performed every two years to check for nephrocalcinosis, which can be brought on by excess calcium substitution (10). The side effects of management were assessed based on their clinical evaluation, which included hypercalcemia, hypercalciuria, and nephrocalcinosis. Patients were believed to have hypercalciuria if the calcium/creatinine ratio was above 0.2.

### 2.3 Genetic analysis

Genomic DNA was extracted from the blood of all the recruited patients using the PUREGENE DNA Extraction Kit according to the manufacturer’s instructions (Gentra Systems, Minneapolis, MN). Gene-specific intronic primers were designed to flank the coding exons of the VDR gene (NM_001017535.2) using the Primer3, v.0.4.0, program (http://bioinfo.ut.ee/primer3-0.4.0/). PCR Amplification was performed in a total volume of 25 mL, containing 20 ng DNA, dNTPs, primers, and Hot Star Taq DNA polymerase (Qiagen, Germantown, MD). Purified PCR products were then sequenced with a BigDyeTM, v3.1, Terminator Cycle Sequencing Kit, and products were read using an ABI PRISM 3730 DNA analyzer (Applied Biosystems, Foster City, CA). Sequencing data was analyzed using the SeqMan 6.1 module of the Lasergene software package (DNAStar, Inc., WI, USA). The predicted effect of each variant was studied using Sort Intolerant from Tolerant (SIFT) and PolyPhen-2. In addition, CADD, which aggregates several prediction algorithms and conservation scores, allelic frequencies, clinical information, and additional annotations using accessible databases via ANNOVAR, was calculated for all the identified variants.

### 2.4 Protocol for VDDR type II management

Patients were initially diagnosed based on their clinical, biochemical, and genetic testing. Based on their initial biochemical profile, patients were placed on a 3 months trial of high doses of oral elemental calcium (5-10 grams/day), if their initial serum calcium was normal with or without low phosphorus levels (while they had radiological changes compatible with rickets with high ALP and high PTH) or were admitted to start an IV calcium infusion if their serum calcium was low (i.e. Ca <2.0 mmol/L), especially if patient presented with hypocalcemic seizures. High doses of oral calcium were administered because, in the absence of vitamin D action, the net calcium retention is only about 10%. If the trial failed after 3 months, the patient was shifted to IV calcium infusion.

### 2.5 Intravenous calcium infusion protocol

After inserting a permanent port catheter, we started a daily intravenous calcium infusion of 1500 mg/m^2^/day over 10-12 hours and all subjects received daily oral magnesium sulfate (5–10 mmol) mid-infusion and oral Phosphate (250–500 mg) at the beginning and end of the infusion. Daily calcium infusion continued till the calcium and phosphate levels normalize, which, on average, takes 8 weeks. Some patients needed a longer period of daily IV calcium, reaching 6 months in severe cases with delayed diagnosis. Then, we shift them to intermittent courses of 7-10 days of daily Intravenous calcium 1500 mg/m^2^ over 10-12 hours every 1-3 months (the frequency is based on monthly laboratory tests, the patient would need IV calcium if his serum calcium was less than 2.1 mmol/L. The patient would take a daily high elemental calcium supplement orally at home (5-10 g/day) every 8 -6 hours.

Once the patients improved radiologically (resolved signs of rickets) and biochemically (serum Ca, PO_4_ and ALP normal for their age), the IV calcium was stopped, and they were kept on daily high doses of oral elemental calcium with their biochemical and radiological data monitored every three. months.

### 2.6 Statistical analysis

Data analysis was done using Windows version 17 of STATA software. Continuous data were reported using mean and standard deviation (SD) or median and interquartile ranges if not normally distributed (IQR]. Categorical data were presented in frequencies (n) and percentages (%). The paired t-test was used to compare the SDS height score pre- and post-treatment to evaluate the overall treatment response. A two-sided p-value less than 0.05 was considered significant.

### 2.7 Ethical approval

This study and its data collection have been approved by the Institutional Review Board at King Faisal Specialist Hospital & Research Centre, Saudi Arabia (RAC reference number: 2235518). This study was performed in accordance with the Declaration of Helsinki 1964 and its later amendments. No informed consent is required for retrospective studies.

## 3 Results

A total of the 42 patients with HVDDR-II were recruited, with females being 24 patients (57.1%) and males consisting of 18 (42.9%). The median age of patients was 12.5 years, with an IQR of [8-16]. Thus, the median age at presentation is 15.5 months, with an IQR of [7-38] (**Table 1**). The most frequently reported symptoms are alopecia in 41 patients (97.6%), followed by short stature (height SDS<-2) in 36 patients (85.7%), bowing of legs in 9 patients (21.4%), delayed walking in 6 patients (14.3%), seizures in 4 patients (9.5%), and respiratory symptoms in 1 patient (2.4%) (**Table 2**). A total of 30 patients (71.4%) presented due to a family history of HVDR Type II (one of the siblings), but still, the most presented after the first year of age.

**Table 1:** Background information of study participants (n=42).

Demographics	n (%), median [IQR]
Gender	
Males	18 (42.9)
Females	24 (57.1)
*The current age (in years)*	12.5 [8-16]
*Age at presentation (in months)*	15.5 [7-38]
*Positive Family history*	30 (71.4)
*Consanguinity*	42 (100.0)
*Birth length (cm)*	50 ± 2.4
*Birth weight (kg)*	3 ± 0.3
*On oral calcium*	7 (16.7)
*On IV calcium*	35 (83.3)
Treatment duration, mean ± SD*	6.2 ± 4.4
Age at diagnosis & starting calcium	
≤12 months	9 (21.4)
>12 months	33 (78.6)

*Reported for 9 patients within a treatment duration ranging from (1 month to 15 years). The other 26 patients are still on IV calcium.

**Table 2:** Presentations (n= 42).

Presentations	n (%)
*Alopecia*	41 (97.6)
*Bowing legs*	9 (21.4)
*Delayed walking*	6 (14.3)
*Seizure*	4 (9.5)
*Respiratory symptoms*	1 (2.4)
Short stature*	36 (85.7)

*Defined as height standard deviation score (SDS) less than -2.

As for initial laboratory tests, the PTH and Alkaline phosphatase (ALP) were high in all participants [IQR: 219-350 ng/L], [IQR: 646-1409 U/l], respectively. The levels of Ca and PO_4_ were considerably low [IQR: 1.6-2.1 mmol/L, 0.8-1.3 mmol/L], correspondingly. However, all had high 1,25-(OH)2D3 levels [IQR: 432-479 pg/mL] (**Table 3**).

**Table 3:** Biochemical data at baseline.

Biochemical baseline	IV calcium (n=35)	Oral calcium (n=7)	Overall
*Initial PTH (n=42)* (Ref.: 10.0-65.0 ng/L)	305 [231-375]	278 [143-287]	289 [219-350]
*Initial ALP (n=42)* (Ref.: 142-335 U/l)	944 [646-1652]	656 [376-1003.8]	929.5 [646-1409]
*Initial Ca (n=42)* (Ref.: 2.10-2.55 mmol/L)	1.8 [1.51-2.1]	2.2 [1.97-2.3]	1.9 [1.6-2.1]
*Initial PO_4_ (n=42)* (Ref.: 0.90-1.80 mmol/l)	1.0 [0.8-1.3]	1.1 [0.8-1.2]	1.0 [0.8-1.3]
*1,25 vitamin D (pg/mL) (n=22)*	479 [432-479]	Not done	479 [432-479]

Data were presented as median [IQR].

Although 30 patients (71.4%) of patients had a positive family history of HVDDR-II, the majority 24 patients (78.6%) were diagnosed and treated after 12 months, and only 9 patients (21.4%) were diagnosed and started the treatment early (at 12 months of age and less). Furthermore, 35 patients (83.3%) were on IV calcium, and 7 patients (16.7%) were on oral calcium. In addition, 29 patients (82.9%) of 35 patients who received IV calcium required it every month or two, and 6 (17.1%) required IV calcium every three months. Consequently, the mean treatment duration for patients who completed the IV calcium treatment was 6.2 years (**Table 1**).

The overall response to treatment was evaluated based on the growth parameters and the biochemical data. For the growth evaluation, the SDS height score was improved significantly (p-value: 0.0064) in the first 5 years of treatment, with a mean SDS score of -3.3 to -2.6 (**Table 4**). Interestingly, when comparing the height SDS pre and post-treatment by gender, males reported a statistically significant improvement in height from (-4.0 to -2.3) (P=0.0000) compared to females (-3.1 to -2.8) (P=0.3612). Consequently, thirteen patients completed their growth and treatment with a mean height of 144.6 cm in females and 152.4 in males (**Table 5**).

**Table 4:** Growth evaluation over 5-year follow-up.

SDS height score	Mean ± SD
*At presentation*	-3.3 ± 1.8
*1^st^ year*	-3.2 ± 1.6
*2^th^ year*	-3.0 ± 1.8
*3^th^ year*	-2.9 ± 1.7
*4^th^ year*	-2.7 ± 1.6
*5^th^ year*	-2.6 ± 1.7

SDS score significantly improved for the first 5 years of treatment from -3.3 to -2.6 with a p-value of 0.0064*.

**Table 5:** Growth evaluation data by gender.

Growth data	Males Mean ± SD	Females Mean ± SD
Height SDS		
-Pre-treatment	-4.0 ± 1.4	-3.1 ± 1.9
-Post-treatment	-2.3 ± 1.1	-2.8 ± 2.0
-P-value	0.0000*	0.3612
*Final height (cm) for those who completed their growth (n=13)*	152.4 ± 6.6	144.6 ± 9.9

*indicates statistical significance.

Regarding the biochemical data, the median levels of ALP, Ca, and PO_4_ were considerably improved compared to the baseline, 929.5 to 315.5 U/l, 1.9 to 2.12 mmol/L, and 1.0 to 1.5 mmol/L, respectively. The median PTH levels improved from baseline 289 to 131 ng/L at the last follow-up but were still high (see **Table 6**). Nephrocalcinosis was absent in all patients, as evidenced by annual renal ultrasound scans. Additionally, the calcium-to-creatinine ratio, monitored every 3 months, consistently remained low across all patients.

**Table 6:** Response to treatment.

Response to treatment	PTH (Ref.: 10.0-65.0 ng/L)	ALP (Ref.: 142-335 U/l)	Ca (Ref.: 2.10-2.55 mmol/L)	PO_4_ (Ref.: 0.90-1.80 mmol/l)
*At presentation, (n=42)*	289 [219-350]	929.5 [646-1409]	1.9 [1.6-2.1]	1.0 [0.8-1.3]
*1^th^ year, (n=36)*	259.5 [194.5-382]	808 [486.5-1071]	1.9 [1.7-2.2]	1.2 [0.94-1.4]
*2^th^ year, (n=35)*	250 [142-349]	559 [326-855]	2.0 [1.8-2.2]	1.3 [0.98-1.5]
*3^th^ year, (n=34)*	193.5 [108-331]	429 [259-734]	2.1 [1.9-2.2]	1.4 [1.1-1.6]
*4^th^ year, (n=32)*	179.5 [114.5-276.5]	422.5 [259-709.5]	2.1 [2.1-2.2]	1.3 [1.13-1.6]
*5^th^ year, (n=26)*	131 [77-224]	315.5 [249-574]	2.2 [2.1-2.3]	1.5 [1.1-1.7]

Data were presented as median [IQR].

Ref., reference range; PTH, Parathyroid hormone; ALP, alkaline phosphate; Ca, Calcium; PO_4_, phosphate.

**Figure 1:**
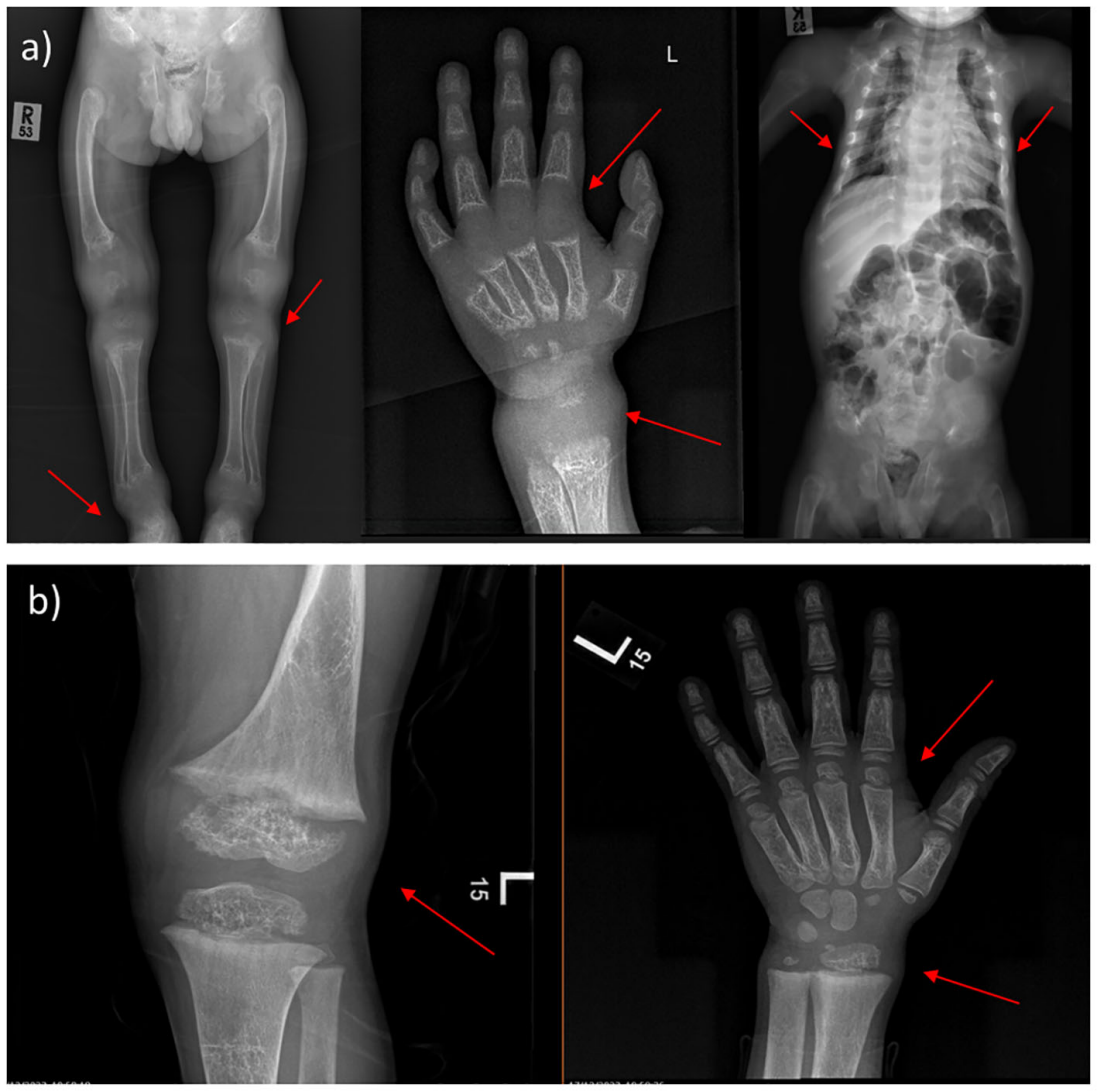
X-ray findings of 4-year-old male. **(A)** Before treatment, the images showing generalized decreased bone density, the profound widening of the growth plate with metaphyseal cupping and fraying of bilateral hips, ankles, knees, and wrists. Narrow chest wall with evidence of significant ricketic changes and costochondral junction fraying. **(B)** One year and a half after treatment (6 weeks IV calcium, then maintained on oral calcium 5 gm every 6 hours daily); showing interval improvement compared to the previously seen growth plate widening and metaphyseal irregularity/fraying, with interval metaphysical sclerosis in keeping with healing rickets. (Case 2 in the **Supplementary File**).

Moreover, the radiological features before and after treatment are shown in (**Figure 1**). After one year and a half on treatment (6 weeks IV calcium, then maintained on oral calcium 5 gm every 6 hours daily), the images showed interval improvement (**Figure 1B**) compared to the previously seen growth plate widening and metaphyseal irregularity/fraying (**Figure 1A**), with interval metaphysical sclerosis in keeping with healing rickets. All patients were confirmed genetically to have HVDDR-II.

**Figure 2:**
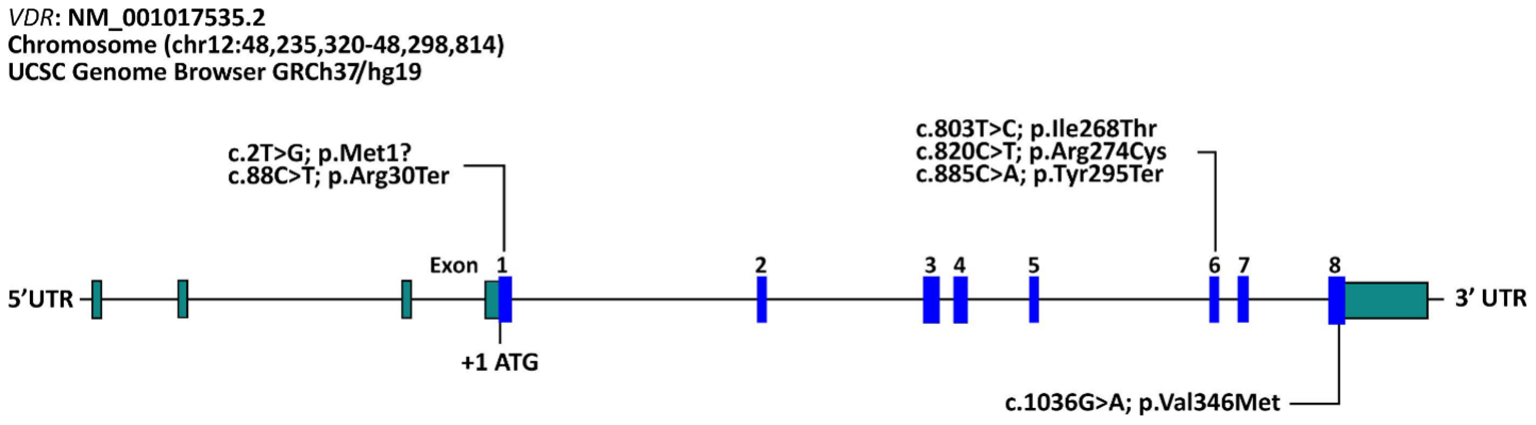
Schematic representation of variants in the *VDR* gene identified in our cohort of Saudi patients with vitamin D-dependent rickets type II. The reference accession number for the *VDR* sequence is NM_001017535.2 and for the encoded protein is NP_001017535.1. Exons of the VDR gene are indicated by boxes and introns by interconnecting lines.

The molecular genetic testing identified six different variants and all variants were Homozygous and already reported before shown in (**Table 7**) as follows: c.885 C>A (p.Tyr295Ter) in 26 patients (61.9%), followed by c.88 C>T (p.Arg30Ter) (n=8; 19.0%), c.1036G>A (p.Val346Met) (n=4; 9.5%), c.820C>T (p.Arg274Cys) (n=2; 4.8%), c.803 T>C (p.Ile268Thr) (n=1; 2.4%), and c.2T>G (p.Met1?) (n=1; 2.4%). The molecular genetic testing identified known Homozygous variants in all patients (**Table 7**, **Figure 2**). The predicted effect of each variant identified in this cohort was found to be pathogenic/disease-causing using *in silico* prediction algorithms tools described in the methodology.

**Table 7:** Variants identified in the *VDR* gene in a cohort of Saudi patients.

HGVS Variant Nomenclature		*In silico* predictions			n (%)	Reference
*VDR*: NM_001017535.2	Genomic position (GRCh37/hg19)	SIFT	POLYPHEN	CADD score		
c.2T>G (p.Met1?)	chr12: 48272895A>C	1 (2.4)	Probably_damaging	25.3	1 (2.4)	Arai et al., 1997 (11)
c.88C>T (p.Arg30Ter)	chr12: 48272809G>A	8 (19.0)	NA	40	8 (19.0)	Mechica et al., 1997 (12)
c.803T>C (p.Ile268Thr)	chr12: 48240544A>G	1 (2.4)	Possibly_damaging	25.7	1 (2.4)	Malloy et al., 2004 (13)
c.820C>T (p.Arg274Cys)	chr12: 48240527G>A	2 (4.8)	Probably_damaging	30	2 (4.8)	Aljubeh et al., 2011 (14)
c.885C>A (p.Tyr295Ter)	chr12: 48240462G>T	26 (61.9)	NA	35	26 (61.9)	Ritchie et al., 1989 (15)
c.1036G>A (p.Val346Met)	chr12: 48238777C>T	4 (9.5)	Probably_damaging	24.4	4 (9.5)	Arita et al., 2008 (16)

NA, not applicable; SIFT, Sort Intolerant from Tolerant; CADD, Combined Annotation Dependent Depletion; HGVS, Human Genome Variation Society.

There was no genotype-phenotype correlation, but it was noticed that three out of four patients with the gene variant c.1036G>A (p.Val346Met) responded to relatively high doses of oral calcium compared to other gene variants whom mainly required IV calcium infusion for years. Interestingly, patients with the following variants demonstrated significant improvement in height with treatment: c.88C>T (p.Arg30Ter), c.885C>A (p.Tyr295Ter), and c.1036G>A (p.Val346Met) (P<0.05) (See **Table 8**). Nevertheless, there is a negative correlation between the gene variants and their growth response to therapy (**Table 8**).

**Table 8:** Variants by height SDS score.

Variant *VDR*: NM_001017535.2	No. of patients	Oral calcium (n=7)	IV calcium (n=35)	Mean age at presentation	Height SDS at baseline	Height SDS at last follow-up	P-value
c.2T>G (p.Met1?)	1	0	1	36 months	-4.0	-1.2	Not applicable
c.88C>T (p.Arg30Ter)	8	0	8	22.6 months (SD: 29.5)	-3.9	-2.1	0.0001*
c.803T>C (p.Ile268Thr)	1	0	1	1 months	0.9	0.1	Not applicable
c.820C>T (p.Arg274Cys)	2	0	2	8.5 months (SD: 2.1)	-1.2	-0.1	0.3554
c.885C>A (p.Tyr295Ter)	26	4	22	22.8 months (SD: 25.6)	-3.2	-2.1	0.0000*
c.1036G>A (p.Val346Met)	4	3	1	74 months (SD: 49.3)	-4.7	-2.0	0.0023*

*indicates statistical significance.

All our patients present with normal birth weight and length, but after the 1^st^ year of life, they develop growth failures. Our study showed that the two patients with the c.820C>T (p.Arg274Cys) pathogenic variant have normal height at presentation regardless of the age of presentation (see **Table 8**). All our patients had a permanent catheter for calcium infusion, with multiple catheter-related complications developed, such as central line blockage in 27 patients (64.3%), which required changing the porta catheter in most of them. Infection developed in 4 (9.5%) of the cases.

## 4 Discussion

HVDDR-II is a rare autosomal recessive disease in the vitamin D receptor (VDR), with ∼ 100 cases reported worldwide. Most of the reported patients were from the Middle East (17). This article presents the largest cohort study of patients with HVDDR-II with typical clinical, biochemical, and radiological evidence. All of them had alopecia except for one case that shared the same pathogenic variant with another patient with alopecia. Alopecia in HVDDR-II is considered a sign of resistance in both hormonal and therapeutic response (18). In our study, patients continued to have total alopecia, unchanged during the follow-up period.

**Figure 3:**
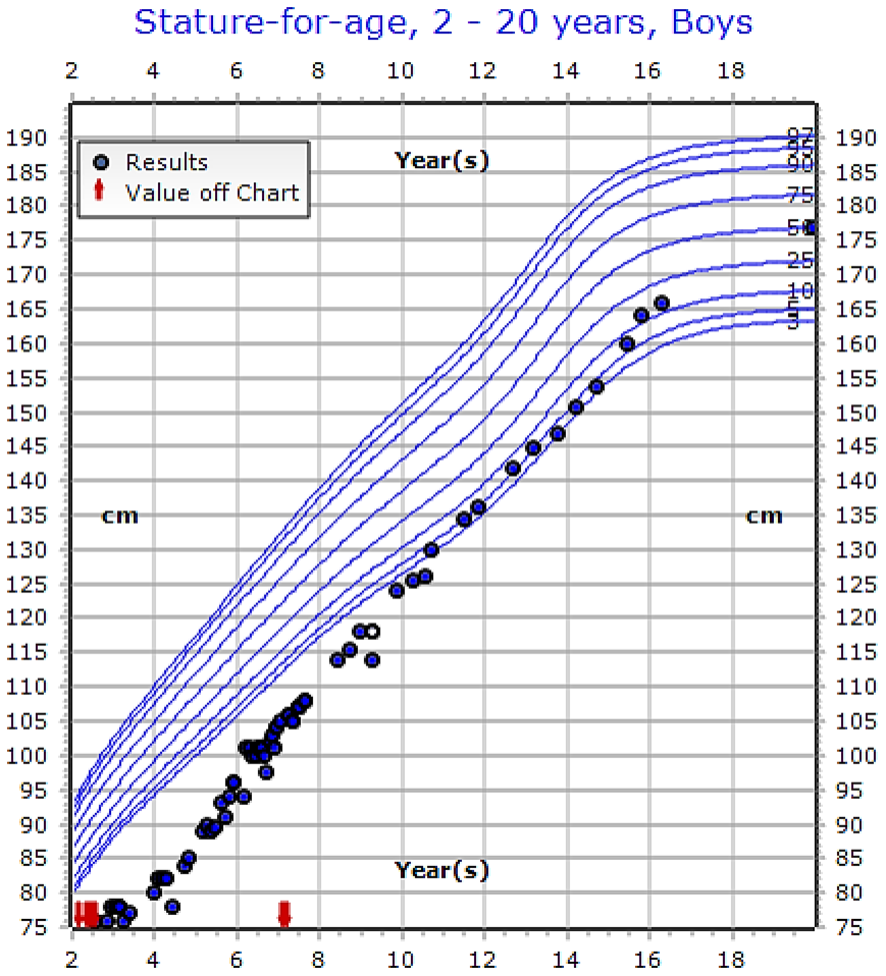
Growth chart of 16-years old male patient showing improvement in his growth with treatment (IV calcium) with genetic variant [c.803T>C (p.Ile268Thr)], (See the **Supplementary Table**, Case 23).

The growth charts of the patients who reached final height showed that the growth velocity improved with the onset of puberty (**Figure 3**). However, male patients had more growth retardation at the onset of presentation in comparison to female patients (see **Table 5**).

The biochemical data at presentation showed that males had low serum calcium levels while females had serum calcium at a lower limit of normal range and elevated PTH levels. Although both genders had the same level of low phosphate and high ALP and marked elevation of 1-25(OH) 2D3 levels, there is a negative correlation between the gene pathogenic variant and abnormal biochemical data.

We found six different variants of *VDR* in our cohort, all of which have been reported before in patients from the Middle East (**Table 7**) (11–16). The most common in our cohort was the c.885C>A (p.Tyr295Ter) variant, followed, in order of prevalence, by c.88C>T (p.Arg30Ter), c.1036G>A (p.Val346Met), c.820C>T (p.Arg274Cys), c.803T>C (p.Ile268Thr), and c.2T>G (p.Met1?).

Previous studies showed that the use of high vitamin D doses could improve the patient’s clinical presentation and biochemical parameters (18, 19). Regarding our study, all patients were given very high doses of vitamin D3 or vitamin D analogs for at least 3 months with no improvement prior to their referral to our hospital. Most of our patients required IV calcium infusion except 7 patients treated with high doses of oral calcium (5-10 grams per day) with good response. Three patients out of four with the (c.1036G>A; p.Val346Met) variant responded to oral calcium, while most patients with the (c.885 C>A; p.Tyr295Ter) variant required IV calcium management for a long duration, suggesting that the type of genetic pathogenic variant may guide the management of this condition.

Studies showed that their serum PTH and ALP levels returned to normal values in patients treated with IV calcium for 5 days monthly after 13 months of treatment. Serial X-ray evaluation demonstrated a dramatic healing of rickets (3). Another study reported that 5 out of 6 patients receiving IV calcium through a peripheral line once a week had improved clinical, biochemical, and radiological characteristics after 2.6 years (20). In our cohort, most patients required IV calcium for at least 5 years and then continued on high doses of oral calcium supplements. Most patients continued to have high PTH despite the improvement of other biochemical and radiological findings, even with frequent IV calcium infusions. All patients who improved and shifted to oral calcium didn’t require IV calcium again. The follow-up of our patients with a median duration of 10 years showed that patients responded very well to the treatment, and height measures improved.

Skeletal manifestation of HVDDR-II can happen due to the involvement of a direct action of vitamin D on the muscle through VDR (21). Furthermore, these manifestations may vary in presentation, and patients with mild defects can be identified during early childhood. The skeletal manifestations include metaphysical cupping and widening, flattening of the skull, dorsal kyphosis, bowing of the legs, and spontaneous remission of the rachitic feature, which can occur between 7 to 15 years when they reach puberty (22). Most of our patients with bone deformities at the onset of presentation improved after enhancing biochemical parameters. In our study, 4 patients out of 13 who have reached their final growth have a mild bowing of the femur, gene varus, and coxa vara, and only 1 patient required referral to orthopedic service for surgical intervention.

Alternative management of HVDDR-II with persistent hyperparathyroidism has been the administration of cinacalcet, a calcimimetic that activates the Ca sensor receptor, enhancing its sensitivity to circulating serum calcium concentrations and thereby decreasing PTH secretion; short term of 2 months administration of cinacalcet to 3 years old with HVDDR enabled relatively long term resolution of clinical, biochemical, and radiographic manifestations of this disease with maintenance therapy of IV calcium infusion (23). We are currently conducting a clinical trial on the use of cinacalcet in cases with HVDDR-II. During the treatment course, our patients were monitored for hypercalciuria and nephrocalcinosis via urinary calcium, creatinine, and renal ultrasound serially every 1-2 years, which showed no hypercalciuria nor nephrocalcinosis seen in any of our patients.

The current study reported valuable insights into VDDR II clinical features and management. However, its retrospective nature limits its generalizability. Therefore, prospective controlled studies are warranted to investigate treatment response in VDDR II patients better.

## 5 Conclusion

This is the largest cohort of patients with HVDDR-II, showing the clinical characteristics and response to current management with the challenges that show that there is still a need for better therapeutic approaches for this population, and these families need genetic counseling for the prevention of recurrence of the disease.

## Data availability statement

The data presented in the study are deposited in the ClinVar repository (https://www.ncbi.nlm.nih.gov/clinvar/), accession numbers: SCV005043784, SCV005043358, SCV005043357, SCV005043355, SCV005043353 and SCV005043351. Further inquiries can be directed to the corresponding author.

## Ethics statement

The studies involving humans were approved by The Office of Research Affairs in King Faisal Specialist Hospital and Research Centre, Riyadh, Saudi Arabia (reference number: 2235518). The studies were conducted in accordance with the local legislation and institutional requirements. The human samples used in this study were acquired from a by- product of routine care or industry. Written informed consent for participation was not required from the participants or the participants’ legal guardians/next of kin in accordance with the national legislation and institutional requirements.

## Author contributions

AA: Conceptualization, Writing – original draft. AA-A: Conceptualization, Writing – original draft. AB: Data curation, Writing – original draft. RA: Formal analysis, Methodology, Writing – original draft. FJ: Data curation, Writing – original draft. KR: Formal analysis, Visualization, Writing – review & editing. FI: Investigation, Methodology, Validation, Writing – review & editing.
